# A single‐cell atlas of cerebrospinal fluid in Alzheimer's disease

**DOI:** 10.1002/alz70855_101801

**Published:** 2025-12-23

**Authors:** Jun Lin, Jiong Shi

**Affiliations:** ^1^ University of Science and Technology of China, Hefei, Hefei, China; ^2^ The First Affiliated Hospital of USTC, Hefei, anhui, China; ^3^ Department of Neurology, The First Affiliated Hospital of USTC, Division of Life Sciences and Medicine, University of Science and Technology of China, Hefei, Anhui, China

## Abstract

**Background:**

Potential differences in immune characteristics between sporadic late‐onset Alzheimer's disease (sLOAD) and sporadic late‐onset AD (sEOAD) have not been well elucidate. Immune cells in the cerebrospinal fluid (CSF) can reflect the brain immune system dysregulation of patients.

**Method:**

Here, we collected CSF of 9 sEOAD patients, 6 sLOAD patients, 11 age‐matched controls for sEOAD, and 10 age‐matched controls for sLOAD from patients in the China Aging and Neurodegenerative Initiative (CANDI) cohort, and performed single‐cell RNA sequencing (scRNA‐seq) and single‐cell TCR sequencing (scTCR‐seq), obtaining a total of 52,719 single cells for further bioinformatics analysis.

**Result:**

We found a reduction in the proportion of T cells and an increase in the proportion of microglia‐like macrophages in the samples of AD patients. Microglia‐like macrophages upregulate inflammation genes in AD patients. The proportion of clonally expanded CSF T cells in sLOAD patients was higher, indicating that antigen‐specific T cells play a greater role in the disease process of sLOAD.

**Conclusion:**

We revealed differences in the transcriptional signatures of immune cells in the CSF of sLOAD and sEOAD. In further studies, we plan to collect the publicly available scRNA‐seq of brain from AD patients with age of disease onset information and combine these with our scRNA‐seq data of CSF for integrated analysis to reveal the potential interactions between immune cells in CSF and brain parenchyma.

